# Design and simulation of the liposomal model by using a coarse-grained molecular dynamics approach towards drug delivery goals

**DOI:** 10.1038/s41598-022-06380-8

**Published:** 2022-02-11

**Authors:** Jalil Parchekani, Abdollah Allahverdi, Majid Taghdir, Hossein Naderi-Manesh

**Affiliations:** 1 Department of Biophysics, Faculty of Biological Sciences, Tarbiat Modares University, Tehran, 14115-154 Iran; 2 Department of Nanobiotechnology, Faculty of Biological Sciences, Tarbiat Modares University, Tehran, 14115-154 Iran

**Keywords:** Computational biology and bioinformatics, Drug discovery

## Abstract

The simulated liposome models provide events in molecular biological science and cellular biology. These models may help to understand the cell membrane mechanisms, biological cell interactions, and drug delivery systems. In addition, the liposomes model may resolve specific issues such as membrane transports, ion channels, drug penetration in the membrane, vesicle formation, membrane fusion, and membrane protein function mechanism. One of the approaches to investigate the lipid membranes and the mechanism of their formation is by molecular dynamics (MD) simulations. In this study, we used the coarse-grained MD simulation approach and designed a liposome model system. To simulate the liposome model, we used phospholipids that are present in the structure of natural cell membranes (1,2-Dioleoyl-sn-glycero-3-phosphocholine (DOPC) and 1,2-Dioleoyl-sn-glycero-3-phosphoethanolamine (DOPE)). Simulation conditions such as temperature, ions, water, lipid concentration were performed based on experimental conditions. Our results showed a liposome model (ellipse vesicle structure) during the 2100 ns was formed. Moreover, the analysis confirmed that the stretched and ellipse structure is the best structure that could be formed. The eukaryotic and even the bacterial cells have elliptical and flexible structures. Usually, an elliptical structure is more stable than other assembled structures. The results indicated the assembly of the lipids is directed through short-range interactions (electrostatic interactions and, van der Waals interactions). Total energy (Van der Waals and electrostatic interaction energy) confirmed the designed elliptical liposome structure has suitable stability at the end of the simulation process. Our findings confirmed that phospholipids DOPC and DOPE have a good tendency to form bilayer membranes (liposomal structure) based on their geometric shapes and chemical-physical properties. Finally, we expected the simulated liposomal structure as a simple model to be useful in understanding the function and structure of biological cell membranes. Furthermore, it is useful to design optimal, suitable, and biocompatible liposomes as potential drug carriers.

## Introduction

One of the methods for studying the theory of the behavior and mechanisms of biological macromolecules is the use of the MD simulation approach^1^. The computer-based method is able to monitor system behavior over time. MD simulation provides information on the structural changes and structural fluctuation of proteins, nucleic acids, membranes, and liposomes^2^. At present, this method is a comprehensive strategy in the studies of behavior, movement, and thermodynamic of biomacromolecules^3^. To run MD simulations, we need the 3D structure of the macro-molecules obtained by X-ray or NMR. The MD simulation could be applied to investigate the stability of proteins, nonbinding modifications, protein folding, and the behavior of molecular complexes, and many other studies^4^. In this study, the formation of a liposome (as a carrier model for drug delivery) was simulated. Liposomes are formed from the accumulation of fat molecules or phospholipids if sufficient energy is expended in the aqueous medium^5^. Liposomes are used as a tool, model, or carrier in basic studies such as cell interactions, drug delivery, food encapsulation, storage and packaging of materials, etc.^6^. Liposomes can be used to trap all three types of hydrophilic, hydrophobic, and amphipathic compounds, as well as prevent the breakdown of trapped compounds and molecules^7^. In general, the use of liposomes as drug carriers has a number of advantages, such as targeting cancerous tissues, increasing the effectiveness and therapeutic index of drugs, increasing drug stability, reducing the toxicity of encapsulated drugs, reducing drug side effects^8^. Furthermore, the biodegradability of the liposome is a very important Indicator in order to drug delivery goals^9^. The stability of the liposome structure must be optimized. One of the factors that affect the stability of liposomes is the type of phospholipids used in the structure of liposomes^10^. Due to the fact that each of the phospholipids has its own chemical-physical properties, their effect on liposomal stability is also different^11^. Therefore, in making a liposome that has good stability, care must be taken not to use any type of phospholipid. Moreover, in the construction of liposomes, appropriate phospholipids with concentration should be used, so that the liposome with optimal stability is obtained. The various phospholipid compounds used in the structure of liposomes, in addition to affecting liposomal stability, also affect the penetration of the drug into the liposome^12^. Liposome formation from different phospholipids in terms of phospholipid content, stability, and cell uptake can be optimized by changing physicochemical parameters^13^. These parameters include bilayer membrane fluidity, surface charge density, surface hydration, and liposome size. Liposomes are considered as a simple model to study the mechanism of membrane structures and membrane-specific functions^14^. Due to the fact that liposomes have a spherical and closed structure, they provide a suitable environment for the packaging and storage of drugs. Due to their structure, liposomes can be used to transport any type of material with different ranges of polarity^15^. As is well known, vesicles are spherically closed structures constituted in water by a double layer of phospholipid molecules. When this surfactant is a phospholipid, vesicles are commonly referred to as liposomes, although often in the literature the two terms are used equivalently. The dimensions of conventional liposomes are typically in the range of 50–500 nm. Liposomes are a very simple model cell membrane, so can be used to study cellular processes and macromolecule function under experimental control^16^. Bunker et al. investigated the application of MD simulation for liposome drug delivery^17^. They successfully described drug loading and release from liposomes, the behavior of liposomes in the bloodstream, and interactions of liposome models with nanoparticle and biological molecules. Lemaalem et al. investigated PEG-coated liposomes by coarse-grained MD simulations^18^. Their results suggest PEG-coated liposomes are the ideal carriers for cancer therapy approaches. While the study of Mahmoudzadeh et al. indicated PEGylation of pH-Sensitive liposomes reduces their efficacy^19^. In this study, we used the coarse-grained MD simulation approach and we created a liposomal model. To simulate the liposome, we used natural cell membrane phospholipids includes of DOPC and DOPE^20^. We were able to identify the role of each of the non-covalent forces in the process of cell membrane formation. In addition, the function of phospholipid and water molecules in this process was also identified.

## Computational method

### Simulated phospholipids

**Figure 1:**
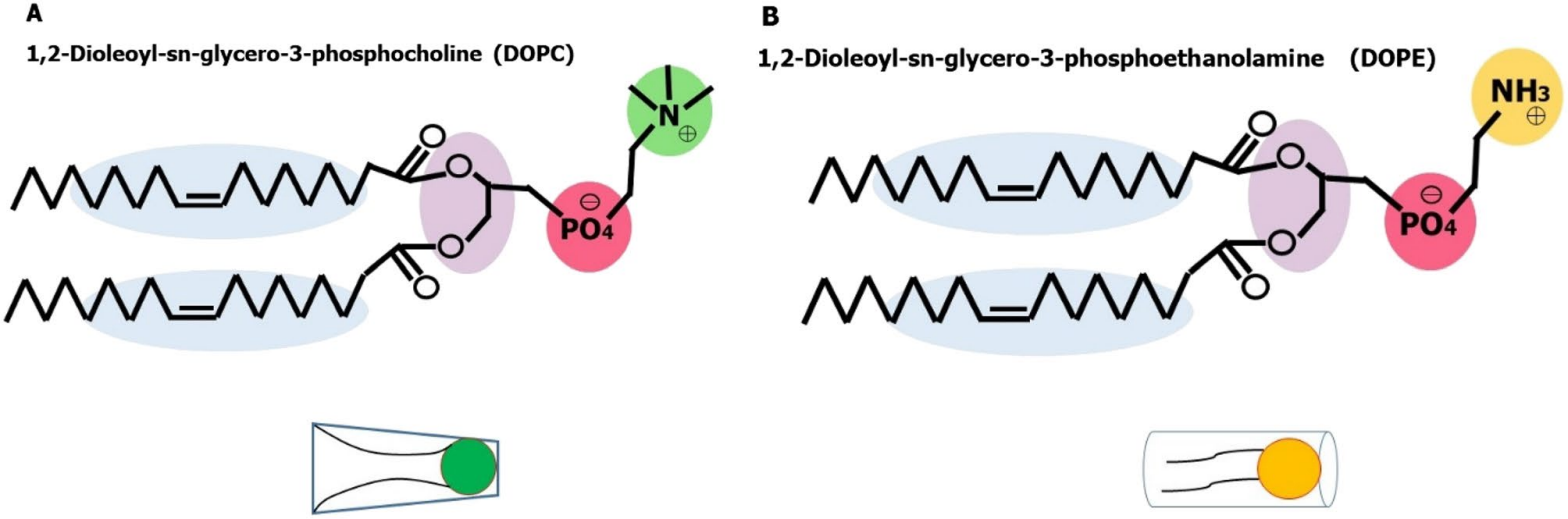
The phospholipids schematic structure used in the system simulation. (**A**) 1,2-Dioleoyl-sn-glycero-3-phosphocholine (DOPC). (**B**) 1,2-Dioleoyl-sn-glycero-3- phosphoethanolamine (DOPE). The glycerol residues are shown in purple, the phosphate groups in red, the fatty acids in blue, the choline group in green, and the amine group in yellow.

DOPC and DOPE phospholipids were selected to create the liposome model. These phospholipids are abundant in the structure of native biological membranes and tend to form liposomes (cells-like). Figure 1 presents the phospholipids schematic structure used in the system simulation. DOPC is a phosphatidylcholine 36:2 in which the phosphatidyl acyl groups at positions 1 and 2 are both oleoyls molecules. This phospholipid derives from oleic acid and is a conjugate base of a 1,2-dioleoyl-sn-glycero-3-phosphocholine. The DOPC phospholipid tends to make the reverse nonlamellar structure^21^. Its geometric structure is conical and has a small head group relative to the core structure^22^. The DOPC consists of two oleic molecules, a phosphatidic acid molecule, a phosphate ion, and a choline molecule. DOPC is used in the laboratory for the generation of liposomes, micelles, and other types of artificial membranes^23^. The second phospholipid used is DOPE, which is a phospholipid present in the structure of biological membranes and its phosphoryl group reacts with ethanolamine molecule. Depending on the length of the fatty acid, the degree of saturation in the C-1 and C-2 sites, and the binding site to the glycerol molecule, glycerophosphoethanolamines can be in different types. The common fatty acids in the structure of phospholipids are 16, 18, and 20 carbons. In the DOPE molecule, ethanolamine is present instead of choline. DOPE is one of the phospholipids with a cylindrical geometric structure that often tends to create a double-layered structure in native biological environments^24^. Real biological membranes are made up of a combination of lamellar and non-lamellar phospholipids and form the structure of double-layered lamellar membranes. Therefore, in this study, we used a combination of two types of lamellar and non-lamellar phospholipids (The cylindrical and conical shape). Finally, we observed the structure of the bilayer membrane as a liposome.

### The coarse-graining of phospholipids

**Figure 2:**
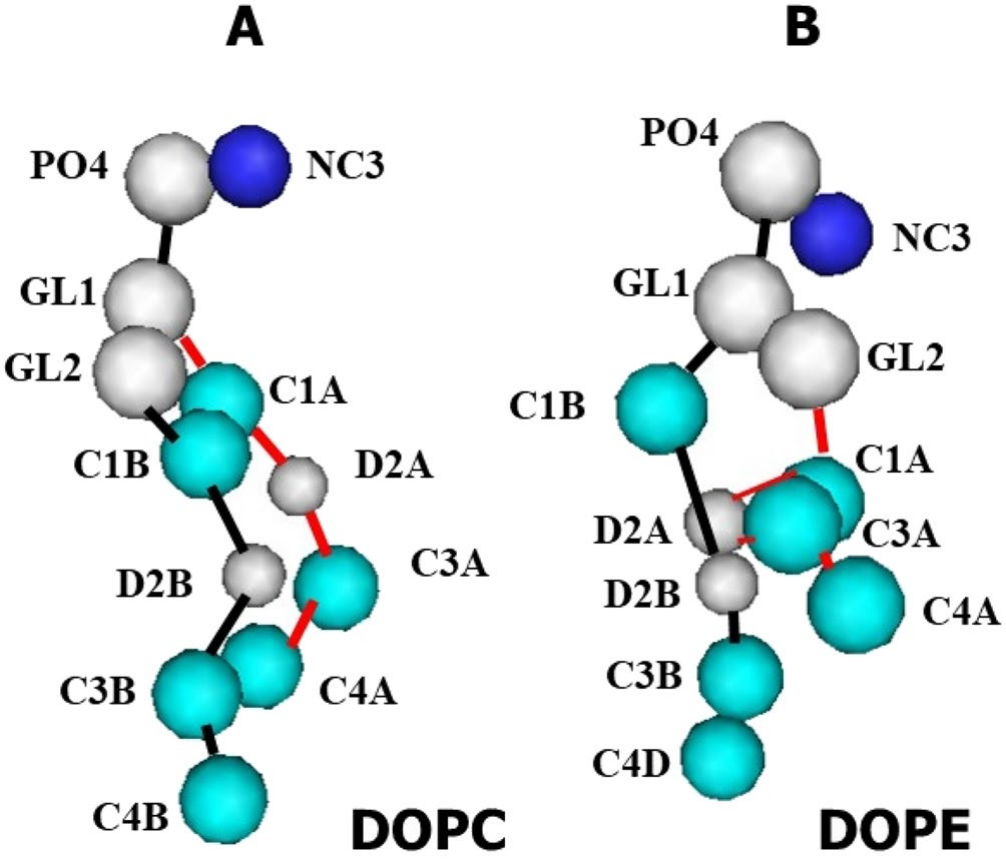
The coarse-grain structure of DOPC and DOPE phospholipids. (**A**) DOPC phospholipid: have beads of NC3 (blue), PO4, GL1, GL2, D2A, and D2B (milky color), C1A, C1B, C3A, C4A, C3B, and C4B (turquoise blue). (**B**) DOPE phospholipid: have beads of NC3 (blue), PO4, GL1, GL2, D2A, and D2B (milky color), C1A, C1B, C3A, C4A, C3B, and C4D (turquoise blue).

The initial data coordinates of phospholipids DOPC and DOPE were obtained as coarse-grained states from the CHARMM-GUI website. Figure 2 presents the coarse-grained structure of DOPC and DOPE phospholipids before being used in the simulation. The simulation of complex biological systems and assembling-based structures such as the bilayer membrane, liposome, mycelium, nanofibers, and other large structures is very long and time-consuming^25^. In this study, the coarse-grained MD simulation approach was used due to the existence of the assembly of phospholipids and needing for a long simulation time. Coarse-grained simulation makes it possible to consider similar nearby atoms as spheres. Chen et al. proposed the coarse-grained MD simulation as an efficient method based that is used to study the physical stability of liposomes^26^. The initial challenge in this study was to select the appropriate phospholipids. It was necessary to select phospholipids that have an appropriate feature for simulating molecular dynamics. The phospholipids that were selected have specific force fields and could be simulated in Gromacs software, version 5.0.1. Therefore, DOPC and DOPE molecules were selected. Each phospholipid molecule (DOPC and DOPE) is prepared as 12 beads. DOPC phospholipid includes NC3 (blue), PO4, GL1, GL2, D2A, and D2B (milky color), C1A, C1B, C3A, C4A, C3B, and C4B (turquoise blue) beads. DOPE phospholipid includes NC3 (blue), PO4, GL1, GL2, D2A, and D2B (milky color), C1A, C1B, C3A, C4A, C3B, and C4D (turquoise blue) beads. It must be noted, the coarse-grained model of lipids, interaction parameters, and the potential function is based on the Martini force field^27–29^.

### Construction of box and adding of water and phospholipids molecules

**Figure 3:**
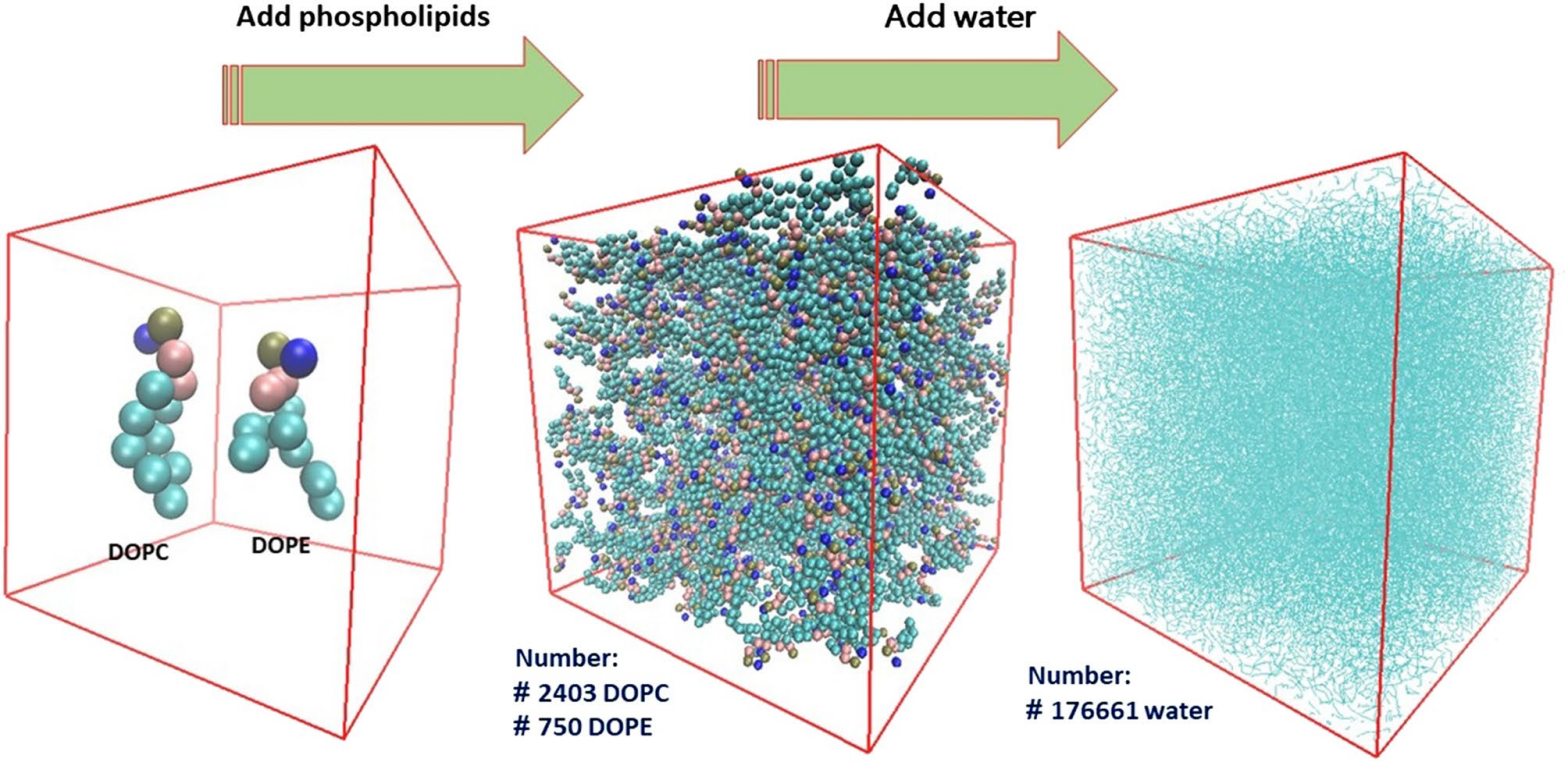
Schematic of simulation box and add phospholipid and water molecules inside the box. The box was made to cubes form and the molecules are homogeneously being distributed throughout the box.

In this study, the conditions of liposome model simulation were prepared using standard protocols and experimental conditions of liposome synthesis (*Epaxal liposome*)^30, 31^. The percentage of phospholipids for forming simulation structure (liposomal model), respectively is 66.66% DOPC phospholipid and 33.33% DOPE phospholipid. In fact, the ratio of DOPC to DOPE (DOPC/DOPE) is 3:1. Initially, in the cubic box with a dimension of 20 nm, 2403 number of DOPC phospholipid molecules, 750 number DOPE, and 176,661 water molecules were added by using Gromacs software package, version 5.0.1. Martini force field (version 2.2) was used out in the simulation of the phospholipid-based system. In the martini force field, the van der Waals and electrostatic interactions potentials are used to define energies. The Martini force field is suitable for studying the behavior of large biological systems such as biological membranes and liposomal models^32^. The parameterization of the force field is based on the thermodynamic properties of DOPC and DOPE phospholipids. The nonpolar water molecule beads were used to solvation of DOPC and DOPE phospholipids. Figure 3 shows the steps for adding DOPC and DOPE phospholipids and water to the simulation box. First phospholipids and then water molecules were added.

### Computational details

Gromacs software package version 5.0.1^33^ was used to performing of all MD simulations. Using the Martini force field (version 2.2), the simulation box was first defined and then phospholipid molecules and water molecules were added, respectively. Then the energy minimization stage of the vesicle pre-structure began. Energy minimization eliminates inappropriate interactions between phospholipid molecules and also provides the appropriate conditions for non-covalent interactions between phospholipid molecules and solvent molecules. This will give the lowest energy to the simulation system. Since at this stage, the biological macromolecules are in a good position alongside the water molecules and there is a proper interaction between these two parts, and the formation of a shelf-like state takes place around the macromolecule. The number of steps for the energy minimization stage, 5000 steps were adjusted. In the equilibration stage, the system pressure was considered constant and equal to atmospheric pressure (1 bar) and the system temperature was set equal to 400 degrees Kelvin. In the equilibrium phase, the stability of the system was controlled in terms of temperature, density, kinetic energy, potential energy, and total energy. The time step was considered to be 20 femtoseconds and periodic boundary conditions (PBC) were applied on all simulations. In this study, one MD simulation process (MD production phase) was performed for the formation of a liposome model, and the simulation time was 2100 ns (2.1 microseconds). The liposome structure was formed by the cooperation of DOPC and DOPE phospholipids and water molecules. Coarse-grained MD simulation is based on considering the coordinates of all grains and requires clear position, velocity, and force parameters between system particles at all times. MD simulations of biological systems, such as liposomal models, are performed to determine the details and mechanism of their formation. Finally, the MD phase result was recorded in trajectory files that containing energy, speed, and location, and necessary analyses were obtained from these files. The analysis was including density, radius of gyration, radial distribution function (RDF), and solvent accessible surface area (SASA), root-mean-square deviation (RMSD), number of clusters, and mean square displacement (MSD). These analyzes were used to investigate the formation mechanism of liposome structure. Moreover, energy analyses were carried out to get the stability of the formed liposome structure.

## Results and discussion

### Liposome formation

**Figure 4:**
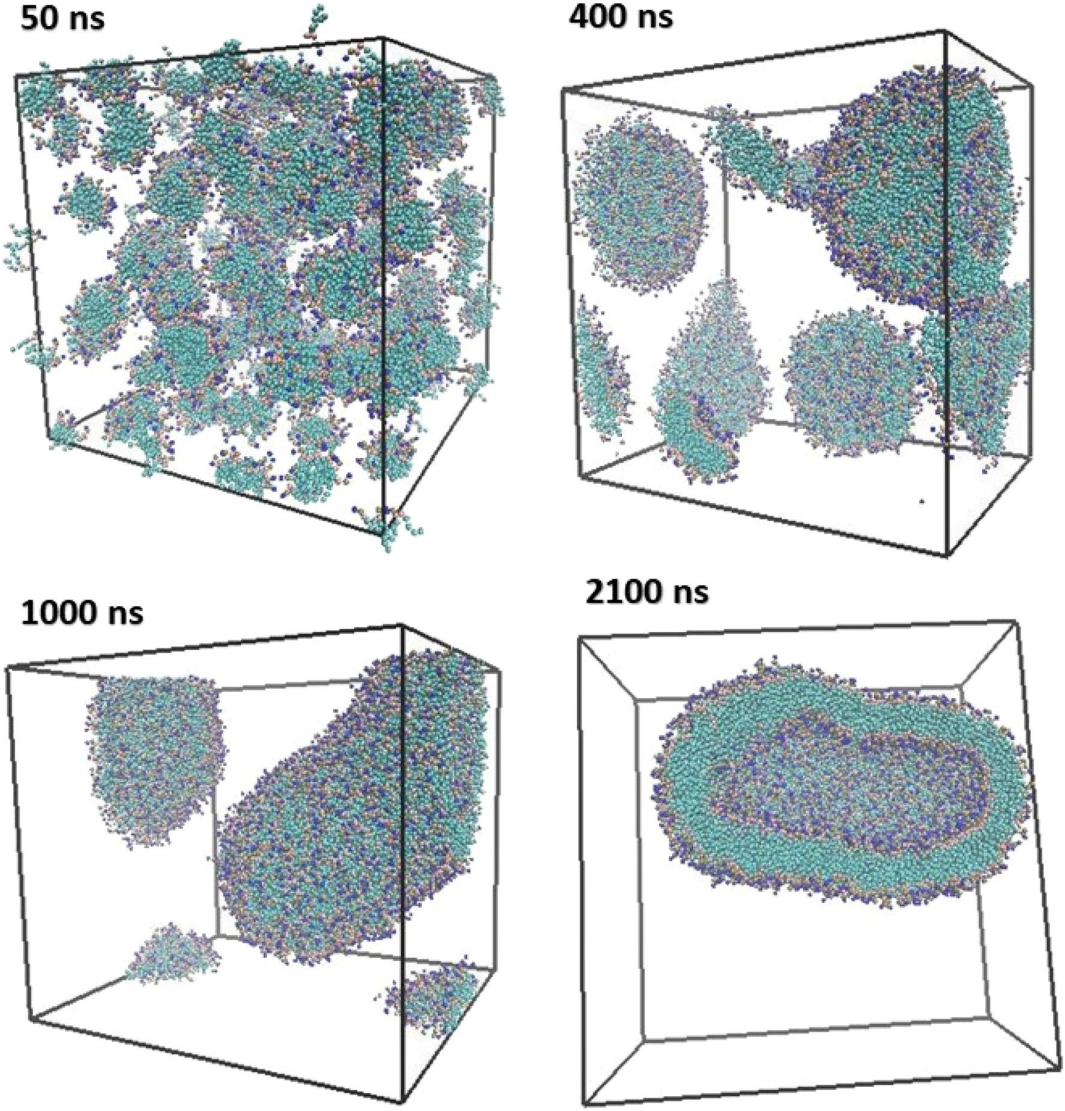
Snapshots of the formation steps of the coarse-grained structure of vesicle from the DOPC and DOPE phospholipids were shown. In the 400 ns and subsequent snapshots, phospholipid particles form a pre-structure of the vesicle. The pre-structure of the vesicle gradually becomes larger with the collision and assembly of phospholipid packs. The times reported that simulation times and water particles were omitted in all snapshots for enhancement of precision. The box is a cubic type with a dimension of 20 nm. Figures are rendered by VMD.

MD simulation is used to study the behavior, structure, dynamics, and mechanisms of different types of biological molecules and macromolecules, such as peptides, proteins, lipids, sugars, solvents, nucleic acids, and etc. The data obtained from the molecular dynamics simulation results with the experimental data, compare and validate. Nowadays, MD simulations are very useful for studying time-dependent processes such as the phenomenon of diffusion, transport, and penetration of materials in membranes^34^. In the study of lipid-based systems such as membranes and liposomes, MD simulation can show the structure, energy, stability, formation mechanism, and interactions and bonds of phospholipids and other system components. In this work, we probed the use of DOPC–DOPE phospholipids molecules to provide the self-assembly process to make a model of liposome structure using coarse-grained MD simulation. The initial structure of the membrane was prepared using standard simulation protocols. Factors such as the degree of lipid saturation, the length of the fatty acid chain, and the lipid head group influence the formation of liposome structure. Energy changes and structural fluctuation of liposomes during the simulation time were monitored using simulation data. The VMD program package (version 1.9.4)^35^ was used to visualize and check the procedure of liposome formation during the simulation runs. At the end of the simulation run (2100 ns), a CG liposome model (vesicle) was formed about 15.6 nm diameter. This structure was the next step for numerical and quantitative analysis. Figure 4 shows snapshots of DOPC–DOPE phospholipids packs that assembled and finally forms elliptical and closed vesicles (a CG structure of liposome model). In fact, one vesicle was formed from the phospholipid concentration. The assembled clumps of phospholipids grow by collision and form nanodisc and bowl-like patches (400 ns snapshot in Fig. 4). Then the phospholipid patches merge together due to more movement and random fluctuation until form an oval and closed liposome (1000 ns snapshot in Fig. 4). Existing forces cause the phospholipids to bend towards inside the liposome lumen and the liposome structure to close. Finally, an adequately large patch forms a liposome model structure (2100 ns snapshot in Fig. 4). Koshiyama et al. investigated the mechanism bicelle-to-vesicle transition of the lipid mixture by coarse-grained MD simulations^36^. They reported that the direction of bicelle bending and vesicle formation is controlled by lipid composition and lipid concentration. Their finding showed the liposome formation respectively have three-phase including bicell formation, bowl formation, and vesicle formation. Although the type and concentration of lipids in our study are different, our findings are consistent with the results of their work, and the vesicle is formed in three stages. However, the advantage of our work is that the type and concentration of lipids are somehow adjusted to form a liposome-like structure (Oval vesicle). Choon-Peng Chng showed the formation of vesicle has a bicelle, distorted bicelle, and bowl-shaped bicelle intermediates^37^. These intermediates were also observed in the simulation process of our study. His result showed the increase of simulation temperature speed up the transition process of the phospholipids to the vesicle. Therefore, we used a temperature of 400 Kelvin, which allowed the vesicle to form properly and quickly.

**Figure 5:**
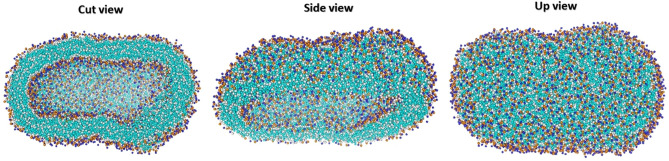
Schematic of the formation of the liposomal model (vesicle) structure in the three different views. Trajectory snapshots from a DOPC–DOPE system that taken after 2100 ns.

The self-assembly process (like a vesicle formation) is an approach during which the components of the system, such as phospholipids, which are in a disordered state and heterogeneously distributed, form an ordered structure by forming local interactions. According to entropic laws, lipids tend to assemble side together in the aqueous medium which this phenomenon is called lipid self-assembly. During the assembly of the lipids, many short-range and long-range interactions are formed and so significant energy is released. Non-covalent interactions include hydrogen bonds and hydrophobic interactions have a critical role in the assembly of lipids^38^. Lipids, depending on their properties and concentrations, can form different types of assembly structures in the water. In our study as expected, a closed and ellipse liposomal structure was formed. Our results showed, due to the nature of DOPC and DOPE structure, a lot number of non-covalent interactions form that have a key role in the formation of the liposomal model. Figure 5 shows the formed vesicle structure. The process of self-assembly to vesicle formation is followed along 2100 ns. The formation of liposomes is a self-assembly process. The width of the liposome bilayer was on the nm scale. The hydrophilic polar head groups of phospholipids formed the outer layers of the membrane, and hydrophobic chains formed the interior layer. The simulation results show that the stretched and ellipse structure is the best structure that can be formed. Like some natural cells and even bacteria that have an ellipse and flexible structure. An ellipse structure is a structure that has less energy than other structures in the simulation condition. The simulation results are compatible with the structure of the native cell membrane that the ellipse structure with its curves on both sides allows intracellular organs and molecules to easily fit inside the cell. It should be noted that liposomes, cell membrane-like structures, and planar bilayer structures are stable structures. Hudiyanti et al. investigated the self-assembly process of DOPE, DLPE, DOPS, DLPS, DLiPS, and DLiPE^39^. Their study showed the three main self-assembled arrangements include planar bilayer, liposome and, deformed liposome there are in the vesicle formation process. They used only one type of lipid in each vesicle structure. However, the advantage of our work is that we used two types of phospholipids (choline and ethanolamine) that have the highest present in the natural membrane structure. Moreover, our structure is a mixture of lamellar and non-lamellar lipids that makes the oval vesicle structure properly formed. Wu et al. investigated the transfer of three drugs with different polarities to the dipalmitoyl phosphatidylcholine (DPPC) and sodium cholate (CHOA) liposome bilayer^40^. Their finding proved the formed vesicle has appropriate compatibility with drug distribution in vesicle structure. Their results confirm that lipids based on the phosphatidylcholine group are suitable for making drug-specific vesicles. Therefore, our vesicle, which has a closed and oval structure and is based on lipids derived from the phosphatidylcholine group, is very suitable for drug delivery. Phospholipids can form many types of assemblies in water (lipid polymorphisms). Many types of agents including hydrocarbon unsaturation, ionic strength, the phase transition temperature of phospholipids, head group size temperature, pH, and etc. modulate polymorphisms of phospholipids. Moreover, the polymorphisms of phospholipids can be modulated by the head group hydration^41^. The short-range repulsive force and an attractive force have a fundamental role in the formation of stable systems such as membranes and liposomes^42^. Orsi et al. used mixed the nonlamellar DOPE lipid and lamellar DOPC lipid to find out the mechanism of many key membrane phenomena. According to this, we used nonlamellar lipid and lamellar lipid in the DOPC 3:1 DOPE ratio. The combination of these two lipids has created a stable vesicle structure^43^. These phospholipids are packed side by side together based on their geometric structure to form an ordered vesicle. The hydrophobic effect that derivate via aliphatic lipid chains is the driving force of the self-assembly of liposomes and cell membrane formation systems. In the next step, the polar head groups of phospholipids play a role in the stability of the self-assembled liposomes. The DOPC and DOPE amphiphilic lipids provide such forces that are needed for stable vesicle equilibrium^44^.

**Figure 6:**
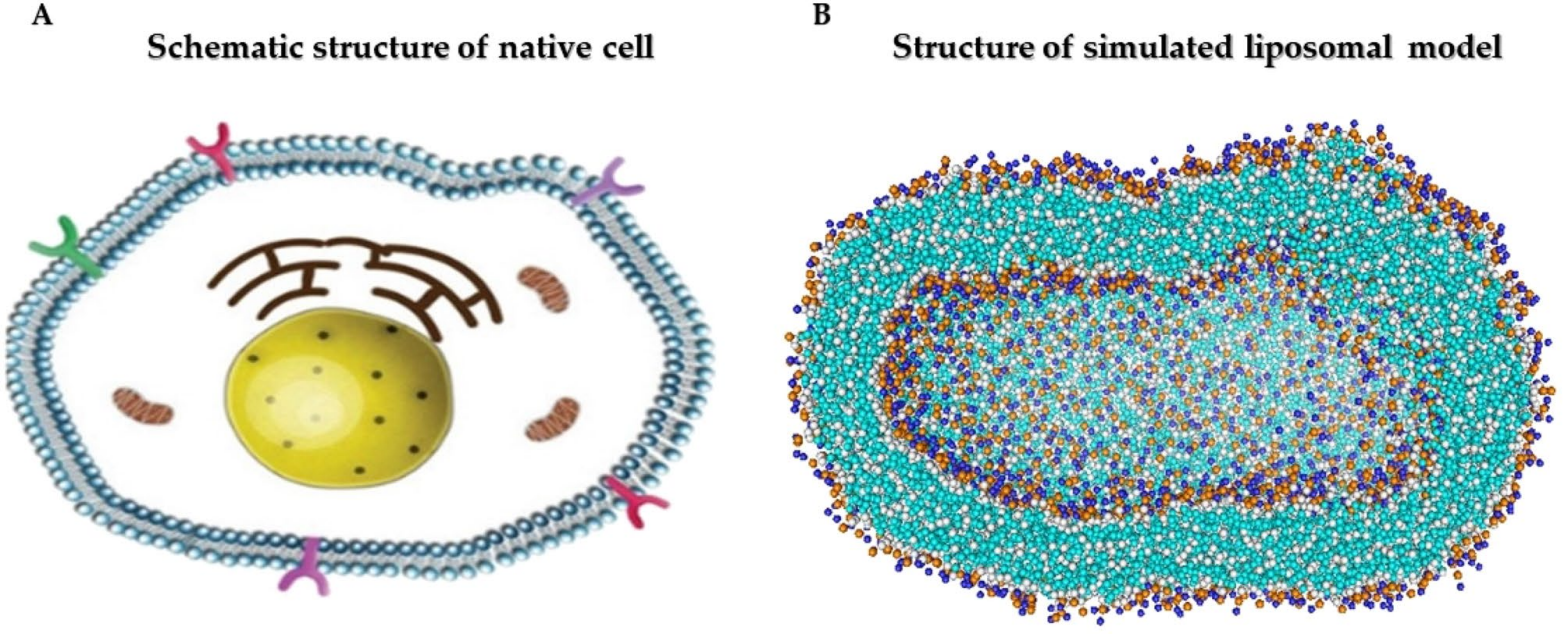
Comparison of the simulated liposome model and native cell structures.

The main shapes of native cells and bacteria are spherical and ellipse structures^45^. The structural geometry and shape of the plasma membrane are very important character that changes in different physiological conditions^46^. This shape change of the cell membrane affects membrane functions such as the transportation of molecules through the membrane, function of the membrane channels, cell absorption, and cell migration. Furthermore, to these applications, cell membranes have an important role in the regulation of cell structure and viscosity^47^. The membrane model (vesicle) that we designed and simulated has a changeable and flexible structure and with these features, it can be considered as a potential model. The structure of the modeled vesicle is a mixture of phospholipids of DOPC and DOPE, which makes the vesicle very suitable for transporting and packing materials. The phase transition temperature (T_c_ (°C)) of DOPC and DOPE phospholipids respectively is − 22 and − 16 °C. Therefore, the modeled vesicle has a flexible and liquid structure. Unlike cells membrane and synthesized liposomal models, there are many different types of fatty acids in the cell membranes of bacteria. Furthermore, bacteria membrane contains unsaturated fatty acids, and modifications such as methylation, hydroxylation and etc. applied on their membrane^48, 49^. Therefore, the simulated liposomal model can’t explain the mechanism of bacterial membranes but is a good simplified model of animal cell membranes. Phosphatidylcholine (45–50%) and phosphatidylethanolamine (15–25%) are the most abundant phospholipids in the cell membrane structure^50^. Thus, these phospholipids were used to get closer to native cell membrane conditions. The simulated liposome model must be able to be as impermeable to charged molecules as biological membranes^51^. Figure 6 shows a comparison of the simulated liposome model and native cell structure. The simulated liposomal models have advantages such as design and synthesis of optimal and low-cost lipid-based carriers, help increase the efficiency of drug carriers, help improve the stability and durability of drug carriers in the patient's body, improve the encapsulation and packaging of materials inside synthetic liposomes and, improve diffusion and the penetration of drugs through the lipid bilayer of the liposome. Liposomal models require validation and accuracy after construction, which shows that successful simulation of liposomes and entering the results into the laboratory is a massive work. Though the design and simulation of Liposomal models are successful, there are still many challenges. Ercan et al. showed membrane properties are affected by oxidation degree of the DOPC phospholipid and by oxidation of the DOPC phospholipid, the permeability of water and methylene blue molecule through membrane increased^52^. We used the native and non-oxidative state of DOPC phospholipid so that the permeability of the liposome model is similar to that of a biological cell. Ding et al. investigated the simulation of five DOPC/DOPE bilayers (lamellar vs non-lamellar lipids) at mixing ratios of 0/1, 1/1, 3/1, 1/3, and 1/0^53^. They prepared membrane bilayers and showed that lipid composition affects membrane function and Chemical-physical properties of the membrane. Since biological bilayer membranes usually contain lamellar and nonlamellar lipids (DOPC/DOPE) at a mixing ratio of 3/1, we constructed a closed-liposome model from DOPC/DOPE lipids with the same features. We hope comprehensive data from the simulated liposome model can be helpful to the construction of whole cell-like models and simulate cellular processes.

### Assessment of vesicle formation and conformational stability

**Figure 7:**
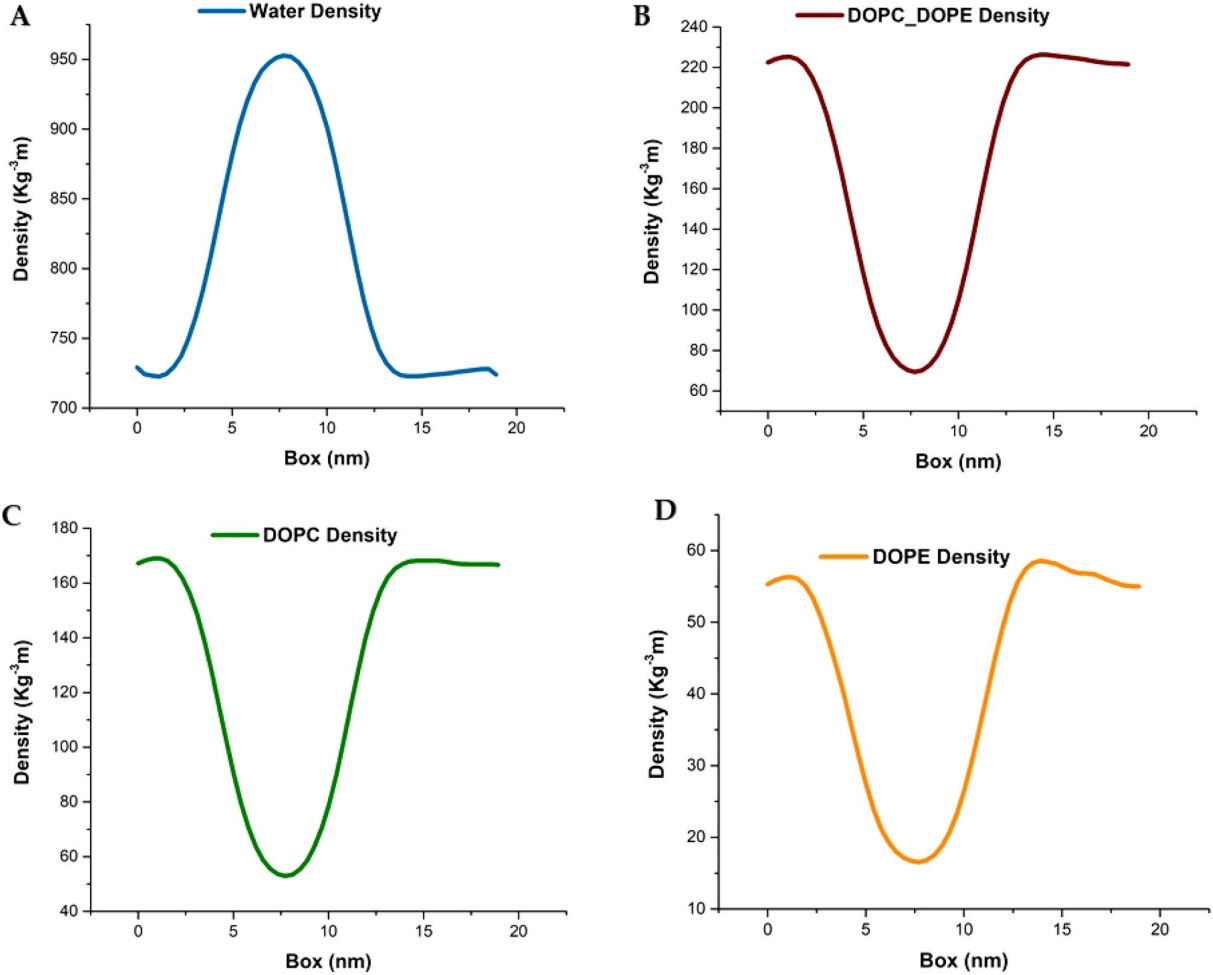
The density profile of the water, DOPC–DOPE, DOPC, and DOPE molecules of the liposome model structure along the simulation box is shown. The density maxima of the water were in the middle of the box (7.5 nm) and the density maxima of the lipids in the margin of the box. These densities confirm that the liposome model structure was formed.

In order to confirm the vesicle formation, the mass density profile parameter is calculated across the simulation box, which shows how the mass of molecules is distributed all over the box dimension. Therefore, the density profiles of DOPC and DOPE phospholipids and water molecules were measured. In a vesicle simulation, the mass density profile is the same shape as the electron density distribution of phospholipid. In fact, the density of the liposomal model is equivalent to the electron density of DOPC and DOPE phospholipids. Moreover, the electron density of all water molecules in the box is considered as the density of water molecules^54^. High density profiles of all atoms (x, y, z dimensions) were measured to check the verification of liposome formation. The procedure of measuring the mass density of molecules is by determining the coordinates of the center of mass of the liposome, and then the coordinates of all the atoms are measured relative to the specified center. Figure 7 shows the water concentrations, DOPC–DOPE phospholipids, DOPC phospholipids, DOPE phospholipids, respectively. According to the curves in Fig. 7, in most same areas of the box, the density of DOPC and DOPE phospholipids and water molecules is reversed. The density of DOPC and DOPE phospholipids in the sides of the box is high while the density of water molecules in the center of the box (9–10 nm) is high. The results of the diagram are perfectly consistent with the structure of the liposome model, given that the lumen portion of the vesicle is in the center of the box and is full of water molecules. Therefore, it is clear in the diagram that in the center of the box, the density of water molecules is high.

**Figure 8:**
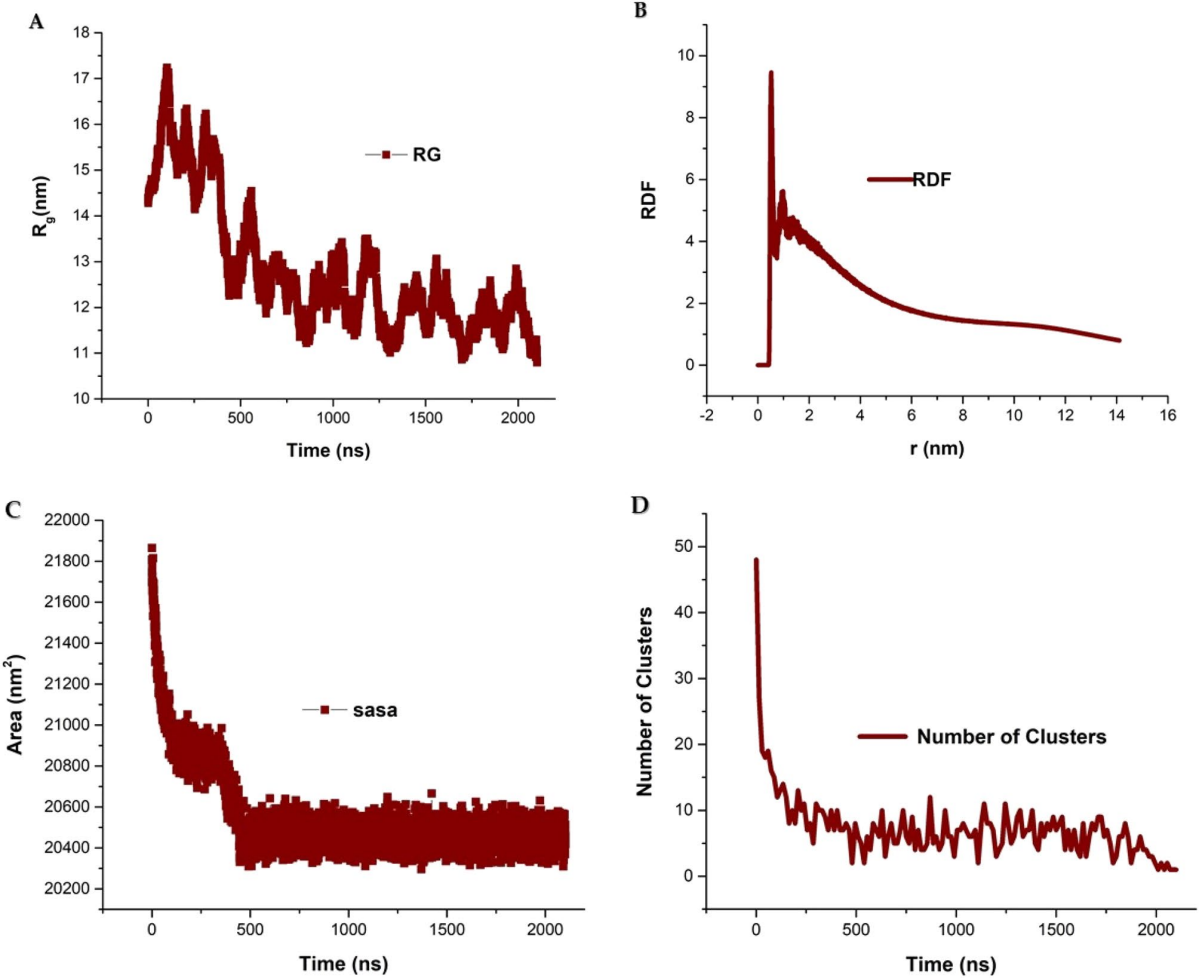
(**A**) The radius of gyration of the liposome structure, which was obtained from the simulated trajectories. The R g value is plotted against the simulation time and decreases during simulation time. (**B**) Radial distribution of PO4 of phospholipids and water particles in the formed liposome structure. The graph indicates the lipids were well-assembled together and made a liposome structure. The high RDF value of phospholipids in the graph indicates a closed and ellipse liposomal model that has formed via phospholipid aggregation. (**C**) Solvent accessible surface area (SASA) analysis of the DOPC–DOPE liposome structure that decreases during the simulation time. This analysis indicates the liposome structure is formed and the lipids are placed side by side. (**D**) The number of clusters of the simulation system. By forming a vesicle structure number of clusters of lipids decreases and at the end of simulation one vesicle formed.

The radius of gyration of phospholipids molecules is the square mean distance of all atoms of the DOPC and DOPE molecules from the axis of rotation of the phospholipid molecule. Moreover, the radius of gyration is a criterion of the compatibility and assembling of phospholipid molecules and an index of the formation of systems like vesicles, liposomes, and micelles. Since the radius of gyration is the distance from the axis of rotation, naturally the value of the radius of gyration index decrease by assembling of DOPC and DOPE phospholipids and formation of the liposomal model during the time^55^. At the beginning of the simulation run, DDOPC and DOPE phospholipids are randomly and heterogeneously dispersed in the water medium. As the simulation time elapses, the phospholipid molecules side by side is located due to their hydrophobicity properties. Therefore, at the end of the simulation, the phospholipid molecules have the lowest value of the radius of gyration. In fact, by measuring the parameter of the radius of the gyration, the three-dimensional structure of the liposome was explored^56^. The radius of gyration of the liposomal model was measured by three-dimensional coordinates of all DOPC–DOPE phospholipids with the presence of water molecules. Figure 8A shows the curve of the radius of gyration of the liposomal model, in which the liposome radius of gyration was noticeably reduced. The relative reduction of the gyration radius is about 6 nm which is significant and confirmed the ellipse and closed liposomal model is formed. These values indicate that lipids have been assembled side together and a closed liposomal structure has been formed.

To investigate the formation of the liposomal model and check the assembling of DOPC and DOPE phospholipids, the RDF parameter was measured. To calculate RDFs, certain atoms such as phosphate oxygen in DOPC and DOPE phospholipids are considered reference atoms^57^. In the next step, the distance and distribution of neighbor atoms toward the reference atom are measured^58^. In the RDF graph, the vertical axis represents the RDFs index, and the horizontal axis represents the distance of atoms of DOPC and DOPE phospholipids from the reference atom^59^. Figure 8B shows the RDF graph for the simulated liposome model structure. The graph shows the two separate peaks that the large peak indicates DOPC phospholipids RDFs and the small peak is RDFs of DOPE phospholipids. Since the concentration of DOPC lipid is higher, this finding is logical. The RDF diagram confirms the DOPC and DOPE phospholipids are in a regular arrangement and have a suitable van der Waals situation. Sometimes the concentration and density of molecules or atoms can be measured by using analysis of the height of the RDF peak. The RDF parameter confirmed the DOPC and DOPE phospholipids are sufficiently ordered and equilibrated and the 2100 ns time scale is a proper time for formation and equilibration of liposomal model structure.

Another parameter that well explains the formation of the liposomal model is solvent accessible surface area (SASA) analysis. The SASA commonly is referred to as the surface of molecules such as phospholipid molecules that are exposed to solvent (water) molecules. The unit of measurement of the SASA parameter usually is the square angstrom, which is compatibles with the dimensions of the molecules under study^60^. A conventional numerical method used to measure SASA is the Shrake-Rupley algorithm method, in which a network of multiple points is drawn at equal distances from the molecule, and then the value of the available surface area to the solvent is calculated^61^. To measure the available surface area, a small spherical ball with a radius of 1.4 Å is rolled on the surface of the molecule, and the solvent accessible surface area, as well as the buried areas, are identified. Naturally, DOPC and DOPE phospholipids have a higher SASA in the monomer and free state than in the liposomal state. The vesicle/solvent interface boundary has been investigated through calculations of the SASA for simulated membranes of DOPC and POPE phospholipids. Moreover, we calculated the SASA of the liposome structure to show how much of its surface is covered by water. We then investigated the dynamics of liposome structure by analyzing the SASA. As shown in Fig. 8C, the value of the SASA decreases during the simulation time, and after 500 ns reaches a constant value. The SASA parameter graph indicates that the liposome structure is formed and the lipids are placed side by side, and the amount of vesicle surface available solvent is reduced. Because lipids are assembled side together, naturally their dynamics decrease during the simulation.

The number of the cluster and packs of lipid was calculated by Clustsize analysis. According to Fig. 4 initially, during the simulation process, DOPC–DOPE lipids spontaneously aggregated, and then they collide together and form plate-like and disc-shaped membranes. The aggregated clumps of DOPC–DOPE phospholipids grow by collision to become bowl-like patches (400 ns snapshot in Fig. 4). Subsequently, the bowl-like patches merge until a sufficiently large one and then form a closed vesicle by bending and curling (2100 ns snapshot in Fig. 4). The number of clusters shows a noticeable difference during the simulation time. Figure 8D represents the number of clusters of liposome model structure which indicates the self-assembly and aggregation of the DOPC–DOPE lipids and the formation of some initial clusters which merged together to make the final closed liposome model. The number of clusters for the simulation system in the initial of the simulation decrease from 48 to 10 cluster and then gradually similarly decrease from 10 to 1 cluster (Fig. 8D). According to the graph, the number of clusters for the system decreased rapidly at an early stage. The high number of clusters at the initiation of the simulation can be the result of high temperature and increasing the phospholipids collision probability and as a result formation of many phospholipids aggregations.

**Figure 9:**
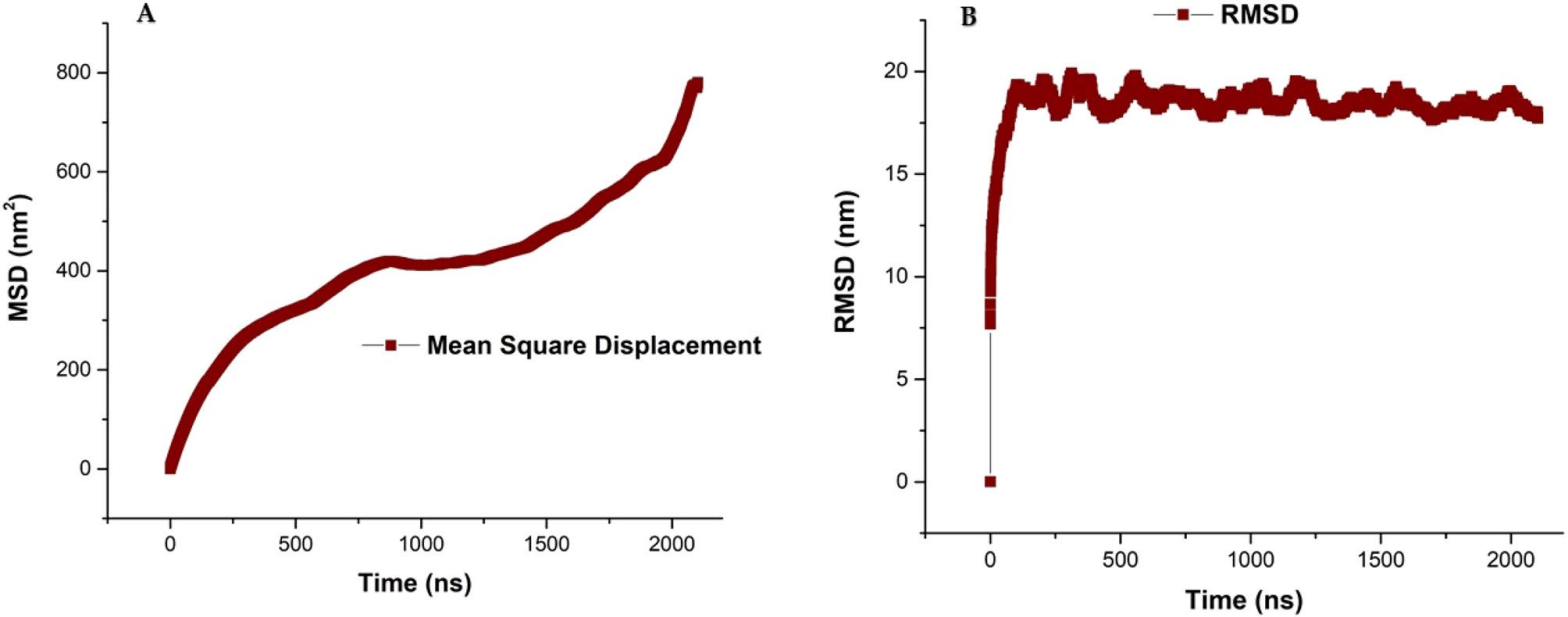
(**A**) The MSD of DOPC–DOPE lipids of the formed vesicle. (**B**) The root-mean-square deviation (RMSD) for DOPC–DOPE lipids of the formed vesicle.

One of the important parameters that can be studied by this method is the measurement of phospholipids lateral diffusion. Therefore, in this study, we tried to calculate this parameter, which is one of the main features of biological membranes. The biological membranes are in a semi-liquid state and the lateral diffusion routinely occurred by phospholipids and protein. Lateral diffusion is a random, spontaneous and, fast movement. In the structure of formed liposomal model, DOPC and DOPE phospholipids have lateral diffusion. With the formation of the final and closed structure of the liposomal model, the value of lateral diffusion of DOPC and DOPE phospholipids in the membrane is expected to increase^62^. Since in our simulated liposomal model only DOPC–DOPE phospholipids exist and there is no restriction, the MSD parameter was calculated. The position of DOPE and DOPE phospholipids in the outer layer or inner layer can be traced and in the next step, the lateral diffusion coefficients for each phospholipid can be measured by using position data. Whatever be more the fluidity of the liposome structure, the lateral diffusion of phospholipids is higher. The calculation of MSD is based on Einstein's relation. In the MSD graph, the slope of the curve is increasing with time and is gaining a reasonable value of the MSD parameter. Lateral displacement of phospholipids does not follow a simple path. This displacement is random and can be considered another form of Brownian motion. Figure 9A indicates the MSD parameter of DOPC–DOPE phospholipids on the 2100 ns time scale. Intramolecular vibrations and motions and rotations of atomic groups around bonds occur on a picosecond time scale. At the picosecond time scale, the motions are very fast and the amplitude of motions is very little. Gradually, over time (the beginning of the nanosecond time scale), the motions of DOPC and DOPE phospholipids increase. Unlike the motion and rotation of hydrophobic tails of phospholipids, the polar head groups of phospholipids have little motion and are almost fixed in place. At the 10 ns time scale, lateral diffusion begins. As shown in Fig. 9A, the lateral diffusion in the nanosecond time scale begins and during simulation time progressed. The presence of cholesterol in the membrane structure and liposomal model reduces the lateral diffusion coefficient of phospholipids. The designed and formed liposomal model in this study is cholesterol-free and contains DOPC and DOPE phospholipids. Therefore, in the constructed liposomal model, a significant value of lateral diffusion motion was observed. Our designed membrane does not have cholesterol content, so lateral diffusion coefficients have the highest possible amount. Although lateral diffusion of phospholipids is measurable in the liquid state of liposomes and membranes but is not measurable in the solid or gel phase. By increasing the temperature of the simulation process, the lateral diffusion of phospholipids increases. Figure 9A shows the MSD parameter for the formed liposome structure, and it is clear that this parameter is increasing during the simulation and with the formation of a liposome model structure. Since this parameter increases as the simulation progress can indicate that the vesicle structure is forming and eventually after the formation of the final “closed liposome model” reaches its maximum value.

The calculation of root-mean-square deviation (RMSD) during the simulation is one of the important indicators in the trajectory of the liposome models. In fact, RMSD shows the degree of deviation of the particle position from the reference position at any point in time. Moreover, the similarity of the three-dimensional structures and structural stability can be measured by using the RMSD parameter. The higher RMSD for one or a group of atoms during the simulation, their structural change during the simulation is great. In other words, the slope of the RMSD diagram indicates the stability of the liposome along the simulation. The closer the slope to zero; the simulated liposome model is more stable. If the slope gradually increases or fluctuates intensely, the simulated liposome model will be more unstable^63^. Figure 9B shows the RMSD parameter for DOPC–DOPE lipids. It is clearly seen that the lipid system attains equilibrium by the formation of vesicle structure and at the end of the simulation system has sufficient stability. The result of Hughes et al. works^64^ suggests the DOPC bilayer has more resistant to harmful effects of the DMSO molecule. Moreover, DOPC phospholipid increases the stability of the membrane systems. The results of the analysis of the energy diagrams of our study show that the DOPC–DOPE based liposomal model has high stability that results from the appropriate interactions of DOPC and DOPE lipids. However, the advantage of our designed and constructed liposome model is that in addition has high stability, it is maybe can proper candidate for drug delivery.

### The effects of non-covalent interactions on the formation and stability of the vesicle

**Figure 10:**
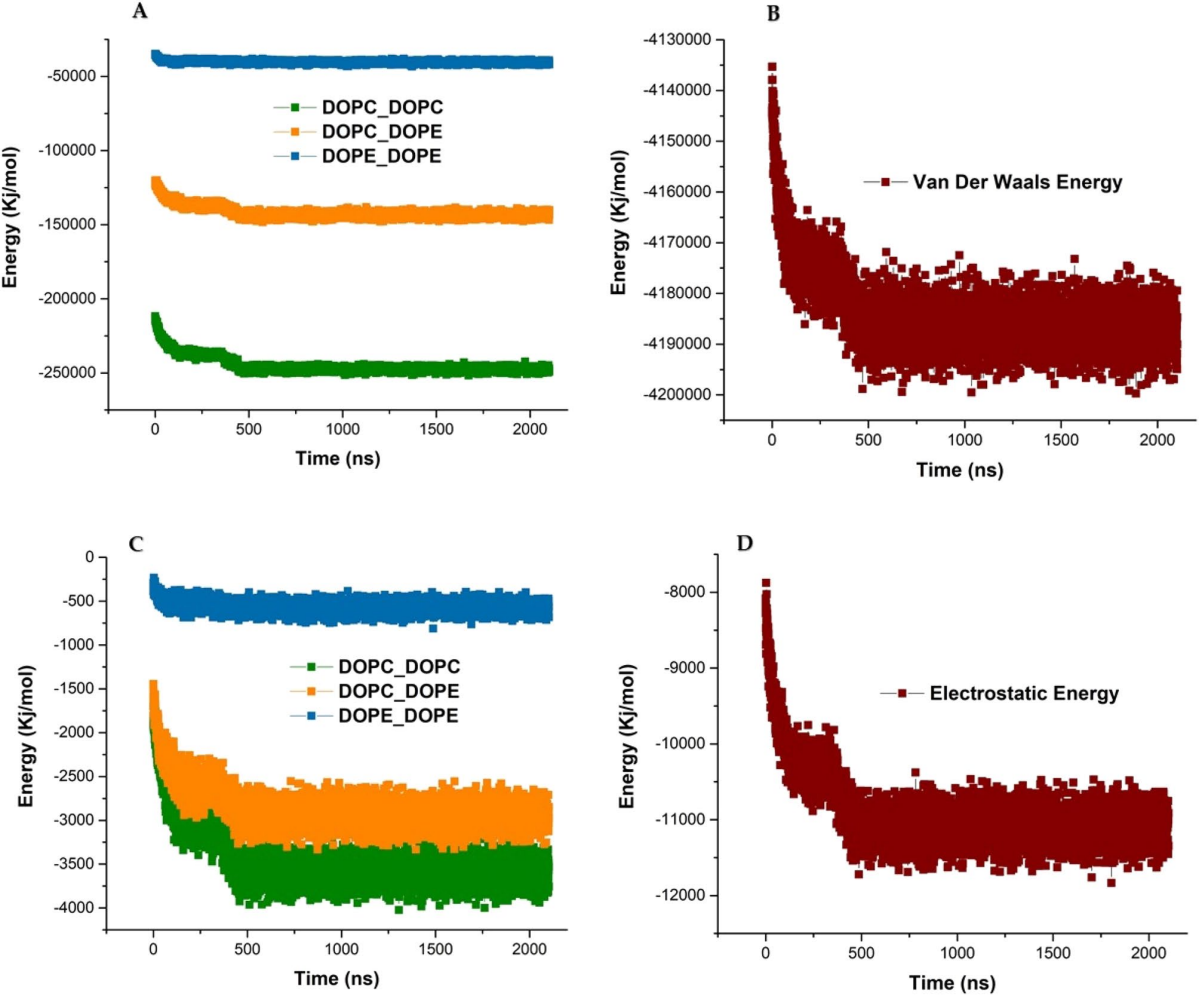
The energy of the van der Waals and electrostatic interaction of the simulated liposome structure. (**A**) Van der Waals interactions of pairs of DOPC–DOPC, DOPC–DOPE, and DOPE–DOPE. (**B**) Total van der Waals interactions of lipids. (**C**) Electrostatic interactions of pairs of DOPC–DOPC, DOPC–DOPE, and DOPE–DOPE. (**D**) Total electrostatic interactions of lipids.

The stability and formation of colloidal particles (liposomes) are directed by the interaction of attractive van der Waals and repulsive electrostatic that is based on the Derjaguin–Landau–Verwey–Overbeek (DLVO) theory. Hydrogen bonding has more role in the initial assembly of phospholipids, while van der Waals interactions in the next steps make more stabilize the structure^65^. The interactions of van der Waals and electrostatic are usually considered short-range interactions. The van der Waals and electrostatic interactions are non-covalent interactions, and although their energy content is low, due to their high number, they have a great impact on the formation and stability of membrane and liposomal structures. The energy content of each van der Waals interaction is from 0.5 to 1 kcal/mol. Besides the interactions of van der Waals and electrostatic have a critical role in the aggregation of DOPC and DOPE phospholipids. In fact, the formation of the lipid-based structure such as liposomes, membranes, micelles, and etc., is mainly directed through non-covalent interactions. The Van der Waals interactions are formed in location between the two layers of the liposome membrane by hydrophobic tails of DOPC and DOPE phospholipids and cause the packing and ordering of phospholipids. It should be noted the van der Waals interactions causing to the formation of bigger clusters of phospholipids. In the simulation condition where the water molecules are considered as beads, the electrostatic and van der Waals interactions drive the liposome model aggregation. Since the number of van der Waals and electrostatic interactions in the structure of the liposome is high, the role of these interactions in liposome formation is very strong^66^. Liu et al. investigated the DOPC membrane showed the CHARMM force field can significantly improve by adjustment of the Lennard-Jones (L-J) parameters^67^. Figure 10 shows the energy of the van der Waals (vdW-Energy) and electrostatic interactions (Coul-Energy) of the simulated liposomal model. The formation and stability of the liposome membrane are mainly determined by the van der Waals interaction energy. Figure 10A shows Van der Waals interactions of pairs of DOPC–DOPC, DOPC–DOPE, and DOPE–DOPE that DOPC–DOPC has the most value of energies. Figure 10C shows electrostatic interactions of pairs of DOPC–DOPC, DOPC–DOPE, and DOPE–DOPE that DOPC–DOPC has the most value of energies. Based on Fig. 10B, D the electrostatic interactions have less role in vesicle formation and stability in comparison with the van der Waals interactions. According to Fig. 10, the van der Waals and electrostatic interactions happen with vesicle formation until they reach a constant value.

**Figure 11:**
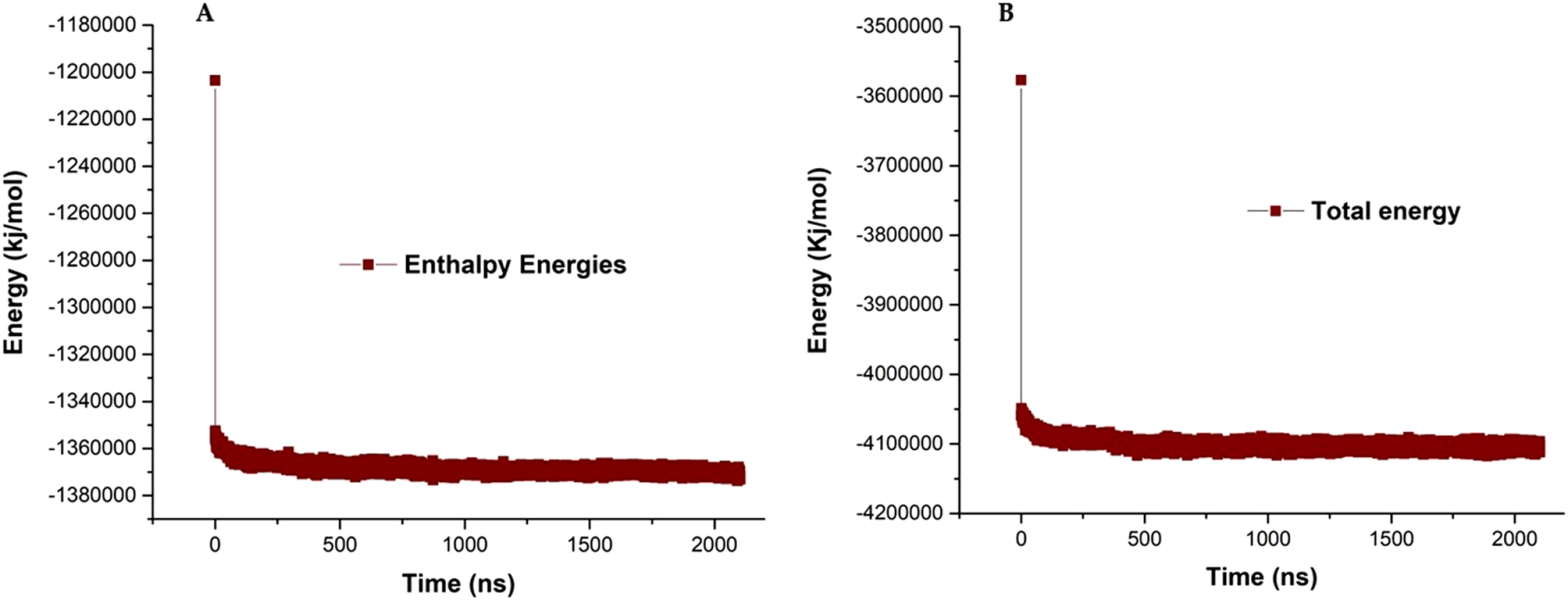
(**A**) Enthalpy energy of liposome model simulation system. (**B**) Total energy of liposome model simulation system.

In addition to the interactions that occur between DOPC and DOPE phospholipid molecules, these phospholipids interact with water molecules. The interaction of liposome phospholipids with water molecules directs the dynamics, flexibility, and stability of the liposomal model structure and also has a significant effect on liposome function. The enthalpy function plays role in the molecular step of liposome formation in terms of solute–solvent interaction. The thermodynamic property of membrane systems is defined by the enthalpy term. In our formed vesicle systems, enthalpy is the sum of the internal energy of DOPC and DOPE lipids. So we tried to get enthalpy energies of the formed liposome model. Figure 11A shows the enthalpy energies of the simulated system. After the passing 20 ns simulation time, the enthalpy energy reaches a constant value. The constant value of enthalpy energy indicates that the structure of the liposomal model has good stability and equilibrium. Figure 11B shows the total energy of the liposome model structure and based on the graph the total energy of the liposome decreased during the liposome formation and reach a constant value. This fact indicates that all of the interactions are suitably formed and the final structure is stable.

Ingram et al. investigated the structure, dynamics, and free energies of membranes and micelles (mixed of DOPC, SOPC, and DMPC lipids) by using combining MD simulations and the COSMO-RS approach^68^. They predicted the free energy profiles of micellar systems and gained worth information about the dynamic, thermodynamic, and kinetic processes of micelles. While we used a mix of DOPC and DOPE lipids that form liposome structures. Our study provides valuable information about the dynamic changes, non-covalent energies profiles, and conformational parameters. However, the advantage of our work can be considered to make a liposomal model that is like natural membranes of lamellar and non-lamellar lipid composition and can be very useful in understanding the function of natural membranes. Risselada et al. using coarse-grained MD simulation studied the effect of membrane composition and curvature effects on the structural and dynamical properties and lipid packing of a liposomal membrane^69^. They speculate that polyunsaturated lipids such as DDPC and DDPE in biological membranes may have an important role in stabilizing curvature regions of liposomes membrane. To check out data with their result, we used polyunsaturated lipids DOPC and DOPE, which cause the proper formation of stable liposome membranes. In addition, the advantage of our study may be that these lipids are abundant in biological membranes and are biocompatible for drug delivery. Magarkar et al. investigated the simulation of PEGylated liposomes with cholesterol molecules^70^. PEGylation increases the circulation time of liposomes in the bloodstream system. Their results showed the structure and compacting of liposome is affected by cholesterol molecules. As a proposed complementary task, we can simulate a liposomal model with a cholesterol molecule in the future. Due to the cholesterol molecule change the compressibility and permeability of liposomes. Tama et al. were used MD simulation to study the self-assembly of eight types of liposomes from four different phospholipids in water^71^. Their results revealed the spherical structure of liposomes is affected by the number of lipids and density of lipids in the inner and the outer layers of liposomes. In our study, the formed liposome structure has different numbers of DOPC and DOPE number of lipids in the inner and the outer layers. It seems different distribution of lipids is needed for the formation of proper and stable liposome models.

## Conclusion

Liposome models are artificial vesicles that are used to investigate cellular processes and macromolecule function under experimental control. The simulated liposome models provide events in molecular biological science and cellular biology. Liposome models have applications in drug delivery, drug packaging, increasing the stability of packaged drugs, targeting specific tissues of the body, gene therapy, helping to formulate substances, intracellular drug delivery. In this study, we used the coarse-grained MD simulation approach to create a liposomal model. To simulate the liposome, we used DOPC and DOPE phospholipids that are abundant in the structure of the natural cell membrane. The simulation results showed one vesicle (ellipse liposome structure) was formed during the 2100 ns. As the natural cell and even the bacterium has an ellipse and flexible structure. Presumably, in these conditions, an ellipse structure is a structure that has more stability than other assembled structures. We were able to get the role of each of the non-covalent forces in the process of cell membrane formation. In addition, the role of phospholipid and water molecules in this process was also identified. Total energy (Van der Waals and electrostatic interaction energy) confirmed the designed ellipse liposome structure has suitable stability at the end of the simulation. The results confirmed that the mix of the nonlamellar DOPE lipid and lamellar DOPC lipid is very appropriate to the liposome formation and finding out the mechanism of vesicle formation. Finally, we expect the simulated liposomal structure as an artificial cell-like model to be useful in understanding the function and structure of biological cells and bacteria. In addition, it is suitable in the design of optimal and biocompatible liposomes as drug carriers.
